# Nocturnal leg cramps: Prevalence and associations with demographics, sleep disturbance symptoms, medical conditions, and cardiometabolic risk factors

**DOI:** 10.1371/journal.pone.0178465

**Published:** 2017-06-06

**Authors:** Michael A. Grandner, John W. Winkelman

**Affiliations:** 1 Department of Psychiatry, University of Arizona, Tucson, AZ, United States of America; 2 Department of Psychiatry, Massachusetts General Hospital, Harvard Medical School, Boston, MA, United States of America; 3 Department Neurology, Massachusetts General Hospital, Harvard Medical School, Boston, MA, United States of America; Associazione OASI Maria SS, ITALY

## Abstract

**Background:**

Nocturnal leg cramps (NLC) are common and poorly understood.

**Objective:**

To determine the prevalence of NLC and associations with cardiometabolic, sleep, and behavioral risk factors in the US population.

**Design:**

Cross-sectional epidemiology.

**Participants:**

National Health and Nutrition Examination Survey, 2005–2006 and 2007–2008 waves.

**Main outcome(s) and measure(s):**

NLC were assessed with, “In the past month, how often did you have leg cramps while trying to sleep?” Responses were categorized as None, Mild, or Moderate-Severe. Demographics, medical history, sleep disturbances, and cardiometabolic risk factors were evaluated using the 2005–2006 dataset. Variables that demonstrated significant relationships to NLC after adjusting for age, sex, education, and BMI were assessed in the 2007–2008 dataset. Variables that were still significant were entered into a forward stepwise regression model combining both waves, to determine which variables best explained the variance in NLC.

**Results:**

Prevalence was 24–25% reporting mild and 6% reporting moderate-severe NLC. NLC increased with age, lower education, unemployment, shorter sleep duration, all assessed sleep symptoms (nocturnal "leg jerks", snoring, snorting/gasping, difficulty falling asleep, difficulty maintaining sleep, non-restorative sleep, sleepiness, use of sleep medications), higher BMI, smoking, medical history (hypertension, heart failure, angina, stroke, arthritis, respiratory disease, and cancer), depression symptoms, and biomarkers (CRP, HbA1c, calcium, cadmium, red blood cells). Stepwise analysis showed that moderate-severe nocturnal leg cramps were associated with (in decreasing order of partial R^2^): leg jerks, poor overall health, arthritis, difficulty falling asleep, age, nonrestorative sleep, red blood cell count, lower education, angina, and difficulty maintaining sleep.

**Conclusions and relevance:**

Based on this first large, representative study, NLC occurring >5x per month are reported by 6% of the adult US population. Sleep disturbance symptoms and health conditions are associated with higher frequency of NLC, suggesting that NLC is a marker, and possibly contributor, to poor sleep and general health.

## Introduction

Nocturnal leg cramps (NLC) are common and their pathophysiology is poorly understood[1, 2]. Their primary morbidity is sleep disturbance and its next-day consequences[3]. Based on small primary care practice surveys[4–6], NLC may occur more commonly with increased age and in those with worse overall health[7]. Other associations with NLC include female sex, leg claudication, electrolyte imbalance, pregnancy, peripheral neuropathy, peripheral vascular disease, angina, and arthritis[8]. Potential medication etiologies include inhaled long-acting beta-agonists, statins, and diuretics[9]. Existing prevalence data suggest that 37–50% of older adults have such leg cramps[4, 5, 10]. In one study in an older population[4], 24% of patients with NLC reported that they were "very distressing". Nevertheless, they are often invisible to clinicians as a minority of patients report them in clinical encounters[5]. An improved understanding of the epidemiology of NLC is a first step in identifying clinically-appropriate methods for recognition and treatment.

To estimate the population-level prevalence of NLC, as well as associations with a wide range of potential correlates, we examined data from the National Health and Nutrition Examination Survey (NHANES). The 2005–2006 NHANES wave was employed as a test sample and the 2007–2008 wave as a validation sample to determine correlates of NLC in a representative US population sample. Hypotheses of the study were that (1) prevalence of NLC increases with age and is more prevalent among women, (2) NLC are associated with other sleep symptoms, (3) NLC are more commonly experienced by individuals with a history of poor health and/or cardiovascular disease, and (4) the report of NLC will coincide with elevated physiologic health risk factors.

## Methods

### Data source

Participants included respondents to the 2005–2006 and 2007–2008 waves of the National Health and Nutrition Examination Survey (NHANES), conducted by the Centers for Disease Control and Prevention[11]. The NHANES data, methodology, and procedures have been previously reported (http://www.cdc.gov/nchs/nhanes). The NHANES is designed to ensure generalizability to the entire US population. Given the complexity of the survey design, coupled with variable probabilities of selection, the data used in the following analyses were also weighted to control for representativeness[11]. Presently, data on adults ages 18–80+ years with complete data were analyzed. All respondents provided informed consent.

### Measures

Full details on measures are reported in freely-available NHANES manuals and on the NHANES website. Variables were chosen if they were present in both the 2005–2006 and 2007–2008 datasets, and have been shown to be associated previously with NLC or could plausibly be related to sleep difficulties associated with leg cramps.

#### Sleep symptoms

NLC were assessed with the item, “In the past month, how often did you have…” One item specified, “leg cramps when trying to sleep?” Responses were categorized as “None,” “Mild” (<15 nights/month), or “Moderate-Severe” (≥15 nights/month). Other sleep variables that were categorized this way included, “trouble falling asleep,” “wake up during the night and had trouble getting back to sleep,” “feel unrested during the day, no matter how many hours of sleep you had,” “feel excessively or overly sleepy during the day,” “take sleeping pills or other medication to help with sleep,” and “leg jerks while trying to sleep.” Snoring and snorting/gasping during sleep (symptoms of sleep-related breathing disorders) were coded as “Never,” “Rarely (1–2 nights per week),” “Occasionally (3–4 nights per week),” and “Frequently (≥5 nights per week).” Typical weekday sleep duration was recorded in whole numbers as a continuous variable.

#### Demographics and socioeconomics

Age was reported in years and categorized in 10-year groups. Sex was self-reported as male or female. Education level was reported as college graduate, some college, high school, or less than high school. Race/ethnicity was coded as non-Hispanic White, Black/African-American, Hispanic/Latino, or Asian/Other. Employment was categorized as yes or no, and typical weekly work hours were coded as a continuous variable. Marital status was reported as married, divorced/widowed/separated, never married, or living with partner.

#### Health history

Overall health was self-rated as excellent, very good, good, fair, or poor. History of hypertension, taking antihypertensive medication, diuretics or long-acting beta agonists, high cholesterol, diabetes, coronary heart disease, heart failure, angina, heart attack, stroke, arthritis, respiratory disease (bronchitis or emphysema), liver disease, thyroid disease, asthma, cancer, and current smoking were self-reported. Depression was evaluated with the Patient Health Questionnaire[12], including total scores computed with and without the sleep disturbance item.

#### Physiologic markers of health

Systolic and diastolic blood pressure were computed based on the average of three readings. Body Mass Index (BMI) was recorded as kg/m^2^. Blood draws were used to estimate levels of cholesterol, creatinine, calcium, potassium, chloride, sodium, folate, c-reactive protein (CRP), iron, ferritin, HbA1C%, glucose, insulin, parathyroid hormone, bicarbonate, vitamins B12, E, and B6, and counts of red and white blood cells. Urine analysis determined levels of cadmium, mercury, and lead.

### Statistical analyses

Univariate comparisons for all variables across severity of nocturnal leg cramps were evaluated using ANOVA or X^2^ as appropriate. The primary analytic strategy was to use the 2005–2006 dataset as a test sample in order to determine which variables should be explored further. To be considered for inclusion in the validation sample (2007–2008), variables needed to demonstrate p<0.05 in the test sample (2005–2006) in a multinomial logistic regression model adjusted for age and sex, with NLC as outcome (either mild or moderate-severe, versus none **All variables that passed this test were assessed in separate models (with mild or moderate-severe NLC outcomes) using 2007–2008 data.** These models were (1) unadjusted, (2) adjusted for age and sex, (3) adjusted for age, sex, education and BMI, and (4) adjusted for age sex, BMI, employment, hypertension, diabetes, and depression. The final model may be useful for examining effects regarding general cardiometabolic morbidity, though it may be over-controlled and thus should be interpreted cautiously. **Finally, since many predictors may explain similar variance, a forward stepwise regression model utilized the combined 2005–2006 and 2007–2008 datasets and included only variables that were significant in age and sex adjusted models in both of these waves, and were not collinear.** This allowed for a wide screen of many potential predictors, refined that list in a second dataset, and further clarified which were the most important in the stepwise procedure. Two-tailed P-values of <0.05 were used. All calculations were performed using STATA/SE version 14 (STATA Corp, College Station TX).

## Results

### Sample characteristics

Weighted characteristics of the analytic samples are reported in Table 1, stratified by sampling wave (2005–2006 or 2007–2008). Prevalence of NLC was consistent across both waves, with approximately 30% reporting symptoms (in both cases, about 24% reported mild and about 6% reported moderate-severe symptoms). Because of the large number of variables originally explored, only those included in the final models are listed. The distributions of covariates are also shown by severity of NLC to explore bivariate relationships among variables using one-way ANOVA or chi-square.

**Table 1:** Characteristics of the sample, stratified by dataset and severity of nocturnal leg cramps.

Variable		Overall Sample	Nocturnal Leg Cramps Severity			
			None	Mild	Moderate-Severe	p
**2005–2006 Sample**						
	%		70.08%	24.21%	5.71%	
Age	≥80	4.18%	3.21%	6.04%	7.35%	<0.0001
	70–79	7.29%	5.96%	9.66%	13.64%	
	60–69	10.71%	10.00%	12.67%	11.20%	
	50–59	17.07%	14.18%	23.62%	25.02%	
	40–49	20.58%	21.52%	18.48%	18.05%	
	30–39	18.36%	20.80%	13.05%	11.12%	
	18–29	21.81%	24.33%	16.48%	13.63%	
Sex	% Female	51.76%	49.74%	54.95%	62.86%	0.0001
Race/Ethnicity	Non-Hispanic White	71.42%	70.26%	74.30%	73.85%	0.1187
	Black/African-American	11.72%	12.24%	10.81%	9.04%	
	Hispanic/Latino	11.44%	12.06%	9.78%	10.68%	
	Asian/Other	5.43%	5.44%	5.11%	6.43%	
Education	College Graduate	26.02%	27.92%	23.59%	13.64%	0.0001
	Some College	31.35%	31.39%	31.34%	31.15%	
	High School	24.94%	23.81%	26.60%	31.40%	
	Less Than High School	17.70%	16.87%	18.46%	23.81%	
Marital Status	Married	55.43%	55.20%	56.70%	53.43%	<0.0001
	Divorced, Widowed or Separated	18.38%	16.48%	20.87%	30.58%	
	Never Married	18.15%	20.30%	14.11%	8.75%	
	Living with partner	8.04%	8.02%	8.32%	7.24%	
Employment	Unemployed	33.07%	29.84%	37.58%	53.01%	<0.0001
Body mass index (BMI)	Continuous	28.5 ± 6.7	28.2 ± 6.7	29.0 ± 6.6	29.5 ± 7.2	0.0014
Smoking	Current Smoker	24.03%	24.26%	21.71%	31.31%	0.0180
Overall Health	Excellent	10.92%	13.02%	7.04%	2.28%	<0.0001
	Very Good	35.58%	38.53%	31.03%	19.52%	
	Good	37.36%	35.30%	42.01%	42.05%	
	Fair	13.95%	11.94%	16.88%	25.64%	
	Poor	2.20%	1.20%	3.04%	10.51%	
Depression	PHQ Score	2.59 ± 3.58	2.19 ± 3.11	3.15 ± 3.85	5.06 ± 5.87	<0.0001
	PHQ Score (-Sleep)	2.06 ± 3.07	1.74 ± 2.66	2.50 ± 3.31	4.11 ± 5.08	
Sleep duration	Continuous	6.90 ± 1.39	6.97 ± 1.34	6.83 ± 1.40	6.41 ± 1.73	<0.0001
Snoring	Frequently	32.35%	30.17%	35.57%	45.32%	<0.0001
Difficulty Falling Asleep	Moderate-Severe	16.01%	12.52%	22.34%	31.95%	<0.0001
Difficulty Maintaining Sleep	Moderate-Severe	20.18%	15.48%	28.20%	43.36%	<0.0001
Non-Restorative Sleep	Moderate-Severe	26.34%	22.92%	31.45%	46.43%	<0.0001
Daytime Sleepiness	Moderate-Severe	18.43%	15.48%	22.91%	35.85%	<0.0001
Leg Jerks	Moderate-Severe	5.85%	2.90%	6.54%	39.15%	<0.0001
Sleep Medication Use	Frequent	8.84%	6.96%	11.60%	20.25%	<0.0001
**2007–2008 Sample**						
	%		68.69%	24.90%	6.10%	
Age	≥80	4.34%	3.74%	5.01%	8.09%	<0.0001
	70–79	7.10%	6.13%	9.10%	9.66%	
	60–69	11.36%	9.63%	14.95%	16.15%	
	50–59	17.65%	15.59%	21.95%	23.26%	
	40–49	19.75%	20.28%	19.08%	16.57%	
	30–39	17.78%	19.79%	13.82%	11.44%	
	18–29	22.02%	24.84%	16.09%	14.84%	
Sex	% Female	48.34%	50.21%	46.96%	32.59%	<0.0001
Race/Ethnicity	Non-Hispanic White	69.22%	68.59%	70.62%	70.67%	0.6796
	Black/African-American	13.42%	13.70%	12.53%	14.08%	
	Hispanic/Latino	11.37%	11.70%	10.87%	9.64%	
	Asian/Other	5.99%	6.00%	5.98%	5.61%	
Education	College Graduate	24.42%	27.18%	20.21%	10.45%	<0.0001
	Some College	20.80%	19.28%	21.37%	36.12%	
	High School	24.75%	23.29%	28.36%	26.34%	
	Less Than High School	30.02%	30.25%	30.06%	27.09%	
Marital Status	Married	56.51%	55.94%	59.66%	49.81%	<0.0001
	Divorced, Widowed or Separated	18.62%	16.56%	21.52%	29.30%	
	Never Married	17.97%	20.14%	13.68%	11.62%	
	Living with partner	6.91%	7.36%	5.14%	9.27%	
Employment	Unemployed	35.98%	32.81%	39.81%	56.24%	<0.0001
Body mass index (BMI)		28.4 ± 6.6	28.0 ± 6.2	29.2 ± 7.3	30.1 ± 8.4	<0.0001
Smoking	Current Smoker	23.46%	22.44%	23.76%	33.81%	0.0003
Overall Health	Excellent	16.84%	19.46%	11.95%	7.53%	<0.0001
	Very Good	31.10%	34.16%	26.89%	13.40%	
	Good	33.72%	33.15%	36.66%	27.58%	
	Fair	14.65%	11.12%	18.78%	38.08%	
	Poor	3.69%	2.11%	5.72%	13.41%	
Depression	PHQ Score	3.12 ± 4.05	2.64 ± 3.51	3.48 ± 4.28	7.03 ± 6.37	<0.0001
	PHQ Score (-Sleep)	2.46 ± 3.48	2.06 ± 3.01	2.73 ± 3.69	5.66 ± 5.61	
Sleep duration	Continuous	6.84 ± 1.39	6.92 ± 1.31	6.75 ± 1.43	6.22 ± 2.02	<0.0001
Snoring	Frequently	31.70%	29.25%	35.47%	44.46%	<0.0001
Difficulty Falling Asleep	Moderate-Severe	18.60%	15.04%	22.65%	42.70%	<0.0001
Difficulty Maintaining Sleep	Moderate-Severe	20.33%	16.79%	23.21%	49.08%	<0.0001
Non-Restorative Sleep	Moderate-Severe	27.68%	23.30%	32.50%	57.82%	<0.0001
Daytime Sleepiness	Moderate-Severe	18.39%	15.36%	20.24%	45.93%	<0.0001
Leg Jerks	Moderate-Severe	6.85%	3.83%	7.36%	39.85%	<0.0001
Sleep Medication Use	Frequent	8.90%	6.97%	10.10%	26.03%	<0.0001

#### 2005–2006 dataset: Variable selection

Regarding the hierarchical regression strategy, all variables were examined using 2005–2006 data to determine inclusion in 2007–2008 models. Results are reported in Table 2. Variables with a significant relationship to NLC were then assessed using 2007–2008 data. The only variable that was retained despite not meeting criteria was race/ethnicity, which was felt to be important to include as a covariate due to its association with other health parameters.

**Table 2:** Relationships between nocturnal leg cramps and variables in the 2005–2006 wave, adjusted for age and sex.

		Mild			Moderate-Severe			Retained for 2007–2008	Included in Stepwise
Variable		OR	95% CI	p	OR	95% CI	p		
Education	College Graduate	1.00	Reference		1.00	Reference		Yes	Yes
	Some College	1.23	(0.98, 1.55)	0.0728	2.12	(1.28, 3.49)	0.003		
	High School	1.28	(1.01, 1.62)	0.0423	2.56	(1.55, 4.25)	0.0003		
	Less Than High School	1.22	(0.97, 1.54)	0.0958	2.66	(1.62, 4.39)	0.0001		
Marital Status	Married	1.00	Reference		1.00	Reference		Yes	Yes
	Divorced, Widowed, Separated	0.98	(0.79, 1.21)	0.83	1.45	(1.02, 2.07)	0.04		
	Never Married	1.00	(0.79, 1.26)	0.97	0.66	(0.41, 1.05)	0.08		
	Living with Partner	1.34	(0.98, 1.82)	0.06	1.24	(0.72, 2.15)	0.44		
Race/Ethnicity	Non-Hispanic White	1.00	Reference		1.00	Reference		Yes*	No
	Black/African-American	0.91	(0.76, 1.08)	0.28	0.78	(0.56, 1.10)	0.16		
	Hispanic/Latino	0.92	(0.75, 1.13)	0.45	1.10	(0.74, 1.62)	0.65		
	Asian/Other	0.98	(0.68, 1.42)	0.92	1.29	(0.68, 2.45)	0.44		
Employment	Yes	1.02	(0.85, 1.23)	0.81	1.89	(1.34, 2.67)	0.00	Yes	Yes
Work Hours	Continuous	1.00	(0.99, 1.01)	0.85	1.00	(0.99, 1.02)	0.80	No	No
Smoking	Current Smoker	1.00	(0.82, 1.22)	0.99	1.82	(1.32, 2.51)	<0.0001	Yes	Yes
Sleep Duration	Continuous	0.91	(0.86, 0.97)	0.001	0.73	(0.65, 0.82)	<0.0001	Yes	Yes
Snoring	Frequent	1.13	(1.05, 1.21)	0.001	1.27	(1.10, 1.45)	0.001	Yes	Yes
Snorting/Gasping	Frequent	1.27	(1.16, 1.40)	<0.0001	1.52	(1.31, 1.76)	<0.0001	Yes	Yes
Difficulty Falling Asleep	Moderate-Severe	1.65	(1.47, 1.84)	<0.0001	1.86	(1.49, 2.33)	<0.0001	Yes	Yes
Difficulty Maintaining Sleep	Moderate-Severe	1.75	(1.57, 1.95)	<0.0001	2.31	(1.85, 2.89)	<0.0001	Yes	Yes
Nonrestorative Sleep	Moderate-Severe	1.59	(1.42, 1.77)	<0.0001	2.30	(1.84, 2.86)	<0.0001	Yes	Yes
Daytime Sleepiness	Moderate-Severe	1.67	(1.49, 1.87)	<0.0001	2.22	(1.77, 2.77)	<0.0001	Yes	Yes
Sleep Medication Use	Moderate-Severe	1.32	(1.16, 1.50)	<0.0001	1.66	(1.37, 2.00)	<0.0001	Yes	Yes
Leg Jerks	Moderate-Severe	2.99	(2.56, 3.51)	<0.0001	6.50	(5.12, 8.25)	<0.0001	Yes	Yes
Overall Health	Excellent	1.00	Reference		1.00	Reference		Yes	Yes
	Very Good	1.46	(1.06, 2.02)	0.02	2.78	(1.11, 6.95)	0.03		
	Good	2.14	(1.56, 2.92)	<0.0001	6.48	(2.66, 15.80)	<0.0001		
	Fair	2.40	(1.69, 3.40)	<0.0001	10.95	(4.47, 26.82)	<0.0001		
	Poor	3.95	(2.22, 7.05)	<0.0001	39.68	(14.43, 109.10)	<0.0001		
Antihypertensive Meds	Yes	1.01	(0.67, 1.52)	0.97	0.82	(0.41, 1.64)	0.58	No	No
Diuretics	Yes	1.00	(0.77, 1.28)	0.973	1.57	(1.08, 2.28)	0.019	Yes	Yes
Long-acting beta agonists	Yes	10.21	(1.22, 85.33)	0.032	3.42	(0.28, 42.18)	0.337	Yes	No
Hypertension	Yes	1.12	(0.93, 1.35)	0.25	1.88	(1.37, 2.59)	0.0001	Yes	Yes
High Cholesterol	Yes	1.18	(0.97, 1.44)	0.10	1.26	(0.90, 1.75)	0.18	No	No
Diabetes	Yes	1.44	(1.10, 1.88)	0.01	1.84	(1.22, 2.78)	0.004	Yes	Yes
Coronary Heart Disease	Yes	1.17	(0.80, 1.71)	0.43	1.57	(0.87, 2.84)	0.13	No	No
Heart Failure	Yes	1.65	(1.09, 2.49)	0.02	2.99	(1.70, 5.26)	0.0001	Yes	Yes
Angina	Yes	1.14	(0.73, 1.76)	0.57	2.01	(1.16, 3.49)	0.01	Yes	Yes
Heart Attack	Yes	1.05	(0.70, 1.57)	0.81	2.38	(1.39, 4.08)	0.0001	Yes	Yes
Stroke	Yes	1.14	(0.75, 1.71)	0.54	1.40	(0.73, 2.71)	0.31	No	No
Arthritis	Yes	1.60	(1.31, 1.95)	<0.0001	2.72	(1.91, 3.86)	<0.0001	Yes	Yes
Respiratory Disease	Yes	1.83	(1.37, 2.45)	<0.0001	2.23	(1.47, 3.38)	<0.0001	Yes	Yes
Liver Disease	Yes	1.18	(0.77, 1.81)	0.44	1.61	(0.85, 3.06)	0.14	No	No
Thyroid Disease	Yes	1.39	(1.06, 1.82)	0.02	1.29	(0.86, 1.95)	0.22	Yes	Yes
Asthma	Yes	1.61	(1.21, 2.14)	0.007	1.83	(1.18, 2.84)	0.01	Yes	Yes
Cancer	Yes	1.13	(0.85, 1.50)	0.40	1.36	(0.88, 2.10)	0.17	No	No
Depression	Score	1.09	(1.06, 1.11)	<0.0001	1.18	(1.14, 1.22)	<0.0001	Yes	Yes
	Score without Sleep	1.09	(1.06, 1.12)	<0.0001	1.20	(1.15, 1.24)	<0.0001	Yes	Yes
Systolic Blood Pressure	mmHg	1.00	(1.00, 1.01)	0.35	1.00	(0.99, 1.01)	0.65	No	No
Diastolic Blood Pressure	mmHg	1.00	(0.99, 1.01)	0.58	0.99	(0.98, 1.01)	0.34	No	No
Body Mass Index	kg/m^2^	1.01	(1.00, 1.03)	0.01	1.02	(1.00, 1.04)	0.02	Yes	Yes
Cholesterol (log)	mg/dL	1.43	(0.97, 2.10)	0.07	1.19	(0.56, 2.54)	0.65	No	Yes
Creatinine (log)	mg/dL	0.91	(0.63, 1.33)	0.63	1.20	(0.64, 2.28)	0.57	No	No
Calcium (log)	mmol/L	0.33	(0.04, 2.85)	0.32	0.00	(0.00, 0.09)	0.002	Yes	Yes
Potassium (log)	mmol/L	1.88	(0.64, 5.55)	0.25	4.30	(0.68, 27.25)	0.12	No	No
Chloride (log)	mmol/L	7.39	(0.38, 145.42)	0.19	0.22	(0.00, 70.29)	0.61	No	No
Sodium (log)	mmol/L	1.11	(0.01, 144.31)	0.97	0.00	(0.00, 1.40)	0.06	No	No
Folate (log)	ng/ml	0.84	(0.71, 0.99)	0.04	0.58	(0.42, 0.79)	0.001	Yes	Yes
C-Reactive Protein (log)	mg/Dl	1.09	(1.02, 1.16)	0.01	1.25	(1.11, 1.40)	0.0003	Yes	Yes
Iron (log)	ug/dL	0.89	(0.73, 1.07)	0.20	0.88	(0.66, 1.17)	0.38	No	No
Ferritin (log)	ng/mL	0.96	(0.81, 1.15)	0.66	1.01	(0.98, 1.04)	0.64	No	No
HbA1c%	%	1.23	(1.12, 1.34)	<0.0001	1.32	(1.17, 1.50)	<0.0001	Yes	Yes
Glucose (log)	mg/Dl	2.16	(1.24, 3.75)	0.01	3.22	(1.18, 8.77)	0.02	Yes	Yes
Insulin (log)	uU/Ml	1.10	(0.94, 1.28)	0.24	1.18	(0.90, 1.54)	0.24	No	No
Cadmium (log)	ug/L	1.02	(0.89, 1.17)	0.79	1.45	(1.09, 1.92)	0.01	Yes	Yes
Mercury (log)	ug/L	0.96	(0.84, 1.09)	0.51	0.93	(0.73, 1.19)	0.55	No	No
Lead (log)	ug/L	0.96	(0.82, 1.13)	0.65	1.02	(0.72, 1.44)	0.91	No	No
White Blood Cell Count	Thousand cells / uL	1.01	(0.98, 1.05)	0.39	1.07	(1.01, 1.13)	0.03	Yes	Yes
Red Blood Cell Count	Million cells / uL	0.99	(0.82, 1.19)	0.88	0.68	(0.48, 0.96)	0.03	Yes	Yes
Parathyroid (log)	pg/mL	0.99	(0.82, 1.19)	0.90	1.03	(0.74, 1.45)	0.84	No	No
Bicarbonate	mmol/L	0.97	(0.93, 1.01)	0.09	0.96	(0.89, 1.03)	0.25	No	No
Vitamin B12	pg/mL	1.00	(1.00, 1.00)	0.08	1.00	(1.00, 1.00)	0.10	No	No
Vitamin E	ug/dL	1.00	(1.00, 1.00)	0.73	1.00	(1.00, 1.00)	0.45	No	No
Vitamin B6	nmol/L	1.00	(1.00, 1.00)	0.18	1.00	(1.00, 1.00)	0.73	No	No

*Although not significant, Race/Ethnicity was retained because it was felt to be conceptually important for 2007–2008 analyses.

#### 2007–2008 dataset: Adjusted models

**Fig 1:**
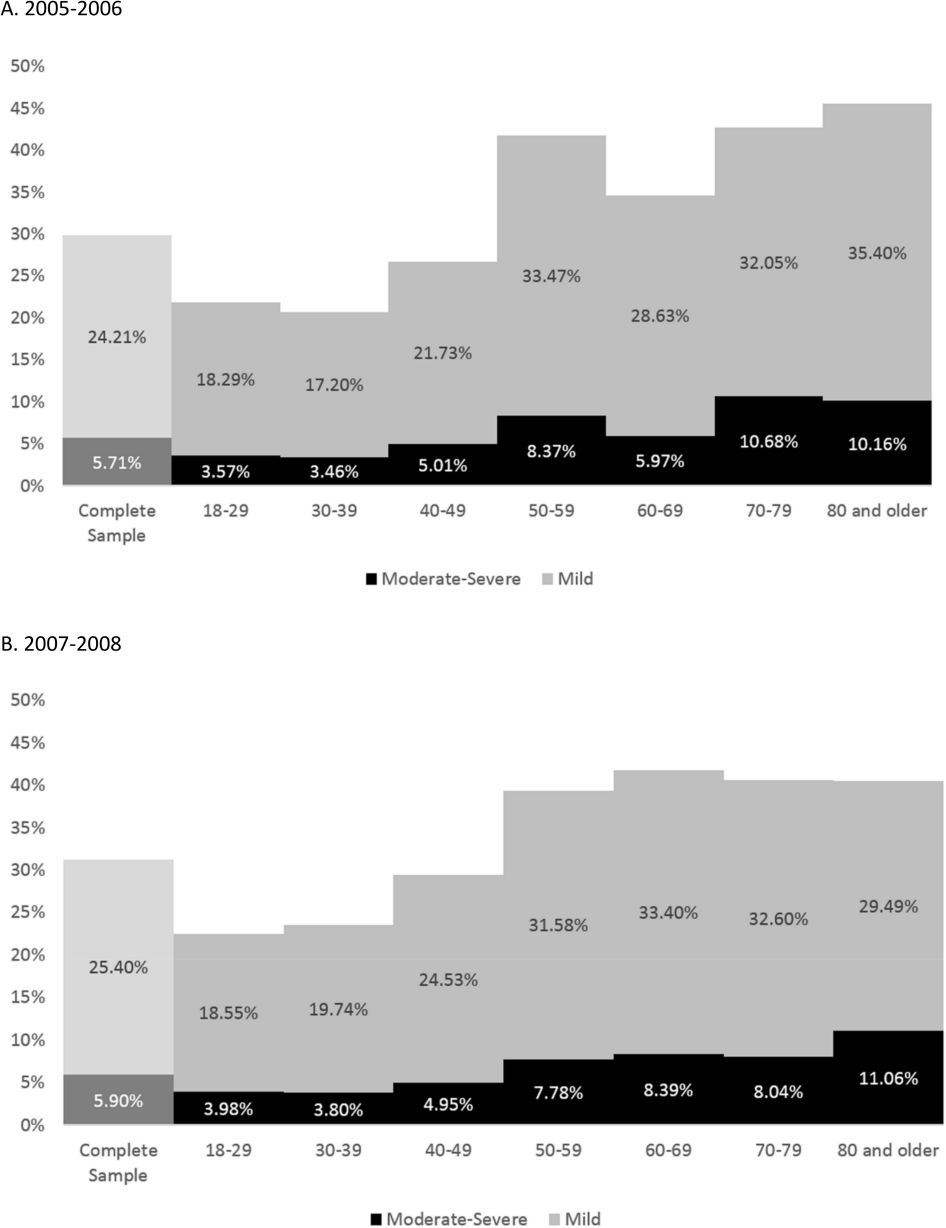
Prevalence of nocturnal leg cramps by age, for 2005–2006 and 2007–2008. (A) 2005–2006. (B) 2007–2008.

Table 3 shows relationships between NLC and demographic and socioeconomic variables retained from the 2005–2006 dataset. In general, prevalence significantly increased with age, assessed continuously (3% increased likelihood per year) and categorically (Fig 1). Women were more likely to report symptoms in both samples. Race/ethnicity differences were seen in the fully-adjusted model, with Hispanics/Latinos reporting fewer leg cramps. Non-college graduates had a higher likelihood of NLC. In addition, unemployment was associated with increased likelihood of reporting NLC.

**Table 3:** Associations with moderate-severe nocturnal leg cramps in 2007–2008 (unadjusted and fully-adjusted models) *.

		Unadjusted			Fully adjusted**		
Variable		OR	95% CI	P	OR	95% CI	P
**Demographics**							
Age	Continuous	1.03	(1.02, 1.03)	<0.0001			
Age	≥80	1.00	Reference				
	70–79	0.73	(0.46, 1.15)	0.172			
	60–69	0.77	(0.50, 1.21)	0.262			
	50–59	0.69	(0.44, 1.08)	0.103			
	40–49	0.38	(0.24, 0.60)	<0.0001			
	30–39	0.27	(0.17, 0.42)	<0.0001			
	18–29	0.28	(0.17, 0.46)	<0.0001			
Sex	Female	2.09	(1.61, 2.71)	<0.0001			
Education	College Graduate	1.00	Reference				
	Some College	4.87	(3.14, 7.57)	<0.0001			
	High School	2.94	(1.83, 4.74)	<0.0001			
	Less Than High School	2.33	(1.45, 3.76)	0.0005			
Race/Ethnicity	Non-Hispanic White	1.00	Reference				
	Black/African-American	0.997	(0.77, 1.29)	0.982	0.82	(0.61, 1.12)	0.211
	Hispanic/Latino	0.800	(0.58, 1.10)	0.166	0.65	(0.46, 0.91)	0.013
	Asian/Other	0.906	(0.52, 1.58)	0.730	1.17	(0.66, 2.08)	0.584
Marital Status	Married	1.00	Reference				
	Divorced, Widowed, or Separated	1.99	(1.50, 2.63)	<0.0001	1.37	(1.01, 1.85)	0.046
	Never Married	0.65	(0.43, 0.99)	0.043	0.86	(0.54, 1.38)	0.540
	Living With Partner	1.42	(0.87, 2.30)	0.163	1.63	(0.97, 2.72)	0.063
Employment	Unemployed	2.63	(2.03, 3.42)	<0.0001			
**Sleep**							
Sleep Duration	Continuous	0.71	(0.6, 0.78)	<0.0001	0.81	(0.73, 0.89)	<0.0001
Snoring	Never	1.00	Reference		1.00	Reference	
	Rarely (1/week)	1.01	(0.63, 1.60)	0.983	1.17	(0.70, 1.97)	0.544
	Occasionally (3-4/week)	1.48	(0.99, 2.21)	0.057	1.53	(0.98, 2.40)	0.063
	Frequently (≥5/week)	2.18	(1.54, 3.08)	<0.0001	1.75	(1.17, 2.64)	0.007
Snorting/ Gasping	Never	1.00	Reference		1.00	Reference	
	Rarely (1/week)	1.88	(1.18, 2.99)	0.007	1.54	(0.92, 2.59)	0.100
	Occasionally (3-4/week)	2.59	(1.70, 3.95)	<0.0001	1.66	(1.03, 2.69)	0.038
	Frequently (≥5/week)	3.68	(2.34, 5.78)	<0.0001	1.89	(1.07, 3.32)	0.028
Difficulty Falling Asleep	None	1.00	Reference		1.00	Reference	
	Mild (<15/month)	1.87	(1.32, 2.66)	0.0005	1.89	(1.28, 2.80)	0.001
	Moderate-Severe	5.98	(4.21, 8.50)	<0.0001	3.39	(2.23, 5.14)	<0.0001
Difficulty Maintaining Sleep	None	1.00	Reference		1.00	Reference	
	Mild (<15/month)	1.93	(1.34, 2.76)	0.0004	1.69	(1.14, 2.50)	0.009
	Moderate-Severe	6.98	(4.89, 9.95)	<0.0001	3.87	(2.57, 5.81)	<0.0001
Non-Restorative Sleep	None	1.00	Reference		1.00	Reference	
	Mild (<15/month)	1.71	(1.16, 2.52)	0.0068	2.32	(1.51, 3.57)	0.0001
	Moderate-Severe	6.36	(4.43, 9.14)	<0.0001	5.59	(3.62, 8.63)	<0.0001
Daytime Sleepiness	None	1.00	Reference		1.00	Reference	
	Mild (<15/month)	2.51	(1.77, 3.56)	<0.0001	2.94	(2.02, 4.26)	<0.0001
	Moderate-Severe	8.58	(6.02, 12.23)	<0.0001	6.06	(4.01, 9.14)	<0.0001
Use of Sleep Medication	None	1.00	Reference		1.00	Reference	
	Mild (<15/month)	1.92	(1.31, 2.81)	0.0008	1.56	(1.01, 2.41)	0.045
	Moderate-Severe	5.10	(3.68, 7.06)	<0.0001	2.71	(1.79, 4.13)	<0.0001
Leg Jerks	None	1.00	Reference		1.00	Reference	
	Mild (<15/month)	3.05	(2.11, 4.43)	<0.0001	2.81	(1.83, 4.32)	<0.0001
	Moderate-Severe	20.3	(4.43, 28.59)	<0.0001	13.42	(8.90, 20.23)	<0.0001
**Medical History**							
Smoking	Current Smoker	1.77	(1.34, 2.32)	<0.0001	1.67	(1.18, 2.37)	0.004
Overall Health	Excellent	1.00	Reference		1.00	Reference	
	Very Good	1.01	(0.53, 1.93)	0.967	0.87	(0.43, 1.74)	0.692
	Good	2.15	(1.21, 3.83)	0.009	1.35	(0.72, 2.56)	0.351
	Fair	8.85	(5.00, 15.64)	<0.0001	4.09	(2.11, 7.91)	<0.0001
	Poor	16.38	(8.71, 30.81)	<0.0001	3.00	(1.38, 6.55)	0.006
Hypertension	Yes	2.86	(2.21, 3.69)	<0.0001			
Diabetes	Yes	3.10	(2.26, 4.26)	<0.0001			
Heart Failure	Yes	3.75	(2.34, 6.00)	<0.0001	1.30	(0.71, 2.39)	0.388
Angina	Yes	2.91	(1.72, 4.92)	0.0001	1.25	(0.67, 2.36)	0.475
Heart Attack	Yes	3.87	(2.58, 5.82)	<0.0001	1.77	(1.07, 2.94)	0.027
Arthritis	Yes	5.59	(4.29, 7.29)	<0.0001	3.66	(2.57, 5.22)	<0.0001
Respiratory Disease	Yes	5.01	(3.59, 6.98)	<0.0001	2.23	(1.52, 3.28)	<0.0001
Thyroid Disease	Yes	2.13	(1.46, 3.11)	0.0001	1.59	(1.04, 2.44)	0.033
Asthma	Yes	3.75	(2.68, 5.25)	<0.0001	2.13	(1.41, 3.20)	0.0003
Depression	PHQ Score	1.19	(1.16, 1.23)	<0.0001			
	PHQ Score—Sleep	1.21	(1.18, 1.25)	<0.0001			
Diuretics	Yes	2.52	(1.84, 3.45)	<0.0001	1.08	(0.69, 1.70)	0.734
Long-acting beta agonists	Yes	4.47	(0.84, 23.70)	0.079	1.84	(0.24, 13.94)	0.055
**Objective Health Variables**							
Body Mass Index		1.04	(1.03, 1.06)	<0.0001			
Calcium	Log	0.27	(0.01, 5.72)	0.4043	1.67	(0.06, 47.37)	0.761
Folate	Log	0.64	(0.52, 0.78)	<0.0001	0.94	(0.81, 1.16)	0.732
C-Reactive Protein	Log	1.34	(1.21, 1.49)	<0.0001	1.14	(0.99, 1.31)	0.074
HbA1c%		1.41	(1.28, 1.55)	<0.0001	1.09	(0.96, 1.25)	0.186
Glucose	Log	3.58	(2.05, 6.24)	<0.0001	1.56	(0.70, 3.51)	0.280
Cadmium	Log	1.40	(1.12, 1.76)	0.003	1.08	(0.83, 1.41)	0.566
White Blood Cell Count		1.10	(1.04, 1.15)	0.0004	1.04	(0.99, 1.10)	0.110
Red Blood Cell Count		0.54	(0.43, 0.70)	<0.0001	0.78	(0.57, 1.08)	0.137

* using variables with p<0.05 in 2005–2006.

** fully adjusted: age, sex, education, BMI, employment, hypertension, diabetes, depression.

NLC were frequently associated with other sleep symptoms. Results are depicted in Table 3 for the unadjusted and fully-adjusted models examining moderate-severe symptoms, S1 Table for analyses across models for mild symptoms, and S2 Table for other models examining moderate-severe symptoms (note that mild and moderate-severe conditions were both included in the multinomial analyses though they are separated in tables for clarity). In all models, moderate-severe NLC were associated with all sleep variables. The relationship to sleep duration was such that every hour of sleep duration was associated with a reduction in the likelihood of moderate-severe NLC by 19%. Further, symptoms of sleep apnea and insomnia were all associated with NLC. The sleep symptom for which the associations were strongest were leg jerks at night.

Results from analyses examining relationships with medical history variables are also reported in Table 3 and S1 and S2 Tables. In the first three models, NLC were associated with poorer overall health, smoking, hypertension, diabetes, heart failure, angina, heart attack, arthritis, respiratory disease, thyroid disease, asthma, and depression. In the final model, no significant associations were seen for heart failure or angina; the rest were attenuated but still significant.

Regarding physiologic health markers, results are also reported in Table 3 and S1 and S2 Tables. Moderate-severe NLC were associated with higher BMI, CRP, HBA1C%, glucose, cadmium, and white blood cell count, as well as decreased folate but not to calcium.

#### 2005–2008 dataset: Stepwise regression

All variables that remained significant after adjusting for age and sex in both models were included in the stepwise model (Table 4).

**Table 4:** Stepwise logistic regression results of associations between nocturnal leg cramps and all significant factors (combined 2005–2008).

		Model Change	Mild Nocturnal Leg Cramps			Moderate-Severe Nocturnal Leg Cramps		
Variable		P	OR	95% CI	p	OR	95% CI	p
Leg Jerks	Mild	<0.0001	3.156	(2.199, 4.531)	<0.0001	3.176	(1.641, 6.147)	0.001
	Moderate-Severe		2.769	(1.567, 4.891)	<0.0001	15.906	(6.887, 36.738)	<0.0001
Overall Health	Very Good	<0.0001	0.748	(0.550, 1.017)	0.064	0.622	(0.277, 1.395)	0.249
	Good		0.77	(0.597, 0.993)	0.044	0.916	(0.532, 1.578)	0.752
	Fair		0.951	(0.758, 1.193)	0.663	0.525	(0.306, 0.899)	0.019
	Poor		2.082	(1.525, 2.842)	<0.0001	3.98	(2.351, 6.737)	<0.0001
Arthritis	Yes	<0.0001	1.667	(1.183, 2.348)	0.003	3.469	(1.897, 6.343)	<0.0001
Difficulty Falling Asleep	Mild	<0.0001	1.387	(0.992, 1.939)	0.056	2.786	(1.428, 5.435)	0.003
	Moderate-Severe		1.9	(1.185, 3.048)	0.008	2.611	(1.179, 5.784)	0.018
Age	Years	0.0003	1.022	(1.012, 1.032)	<0.0001	1.03	(1.010, 1.049)	0.002
Non-Restorative Sleep	Mild	0.0022	1.096	(0.763, 1.575)	0.62	2.052	(1.054, 3.996)	0.034
	Moderate-Severe		1.806	(1.184, 2.753)	0.006	3.846	(1.826, 8.100)	<0.0001
Red Blood Cell Count		0.0069	1.16	(0.859, 1.566)	0.334	0.422	(0.235, 0.756)	0.004
Education	Some College	0.0163	1.653	(1.063, 2.568)	0.026	2.671	(1.249, 5.711)	0.011
	High School		1.645	(1.09, 2.482)	0.018	1.194	(0.513, 2.781)	0.68
	Less Than High School		1.436	(0.957, 2.155)	0.081	1.132	(0.449, 2.854)	0.793
Angina	Yes	0.0214	0.32	(0.123, 0.833)	0.02	0.229	(0.066, 0.801)	0.021
Difficulty Maintaining Sleep	Mild	0.021	1.463	(1.029, 2.080)	0.034	1.082	(0.578, 2.023)	0.806
	Moderate-Severe		0.882	(0.544, 1.428)	0.609	1.462	(0.718, 2.979)	0.295

Of note, categorical variables were dummy-coded, such that each categorical variable produced k-1 dummy variables, where k is the number of categories to reduce collinearity.

In order of partial R^2^, unique contributors to the variance of nocturnal leg cramps were: leg jerks (positive relationship), overall health (positive relationship for poor health), arthritis (positive relationship), difficulty falling asleep (positive relationship), age (positive relationship), non-restorative sleep (positive relationship), red blood cell count (negative relationship), education (positive relationship for only those with some college), angina (negative relationship with moderate-severe leg cramps), and difficulty maintaining sleep (positive relationship only for mild NLC).

Because the relationship with leg jerks was so strong, the stepwise analysis was run excluding the leg jerks variable (See S3 Table) to determine which variables would be related to leg cramps if the variance accounted for by leg jerks was excluded. In this case, the order of variables changed slightly, and asthma and HbA1c% were added to the list (both positive relationships). Since overall health is a non-specific variable, a third stepwise regression analysis was performed excluding both leg jerks and overall health. Results of this model, displayed in S3 Table, shows that depression was added to the list.

## Discussion

This is the first large-scale, population-level epidemiologic investigation of NLC. We found that 30% of adults report having NLC at least 5 times per month, with 6% reporting them at least 15 times per month. These prevalence rates were consistent between the two waves two years apart. As analyses were cross-sectional, causal relationships between NLC and specific medical conditions or biomarkers cannot be established. However, important predictors of frequent NLC were established from these cohorts and were generally consistent between the two waves. It is notable that in the models and the stepwise analyses, the associations with health outcomes were consistently stronger as the frequency of NLC increased, increasing our confidence in the validity of these associations. For example, the relationships with poor overall health and multiple medical illnesses and depression increased in strength as the frequency of NLC increased. Similarly, relationships with NLC were generally stronger for predicting moderate-severe symptoms versus mild symptoms of sleep disturbance.

Consistent with previous studies in small, mostly older populations, advancing age was associated with more frequent NLC[4, 7, 13]. It is unclear which features of aging independently predispose to NLC; however, the association could also be a proxy for medical illnesses which were not assessed in this NHANES wave, such as peripheral arterial disease and/or peripheral neuropathy. Although NLC were more common in women than men, which is consistent with some[13], but not all[4], previous studies, sex was not an independent risk factor in the stepwise regression after factoring in other variables (leg jerks, overall health, arthritis, etc.). No race/ethnicity differences in reports of NLC were observed, though Hispanic/Latino ethnicity was protective in the 2007–2008 cohort after full adjustment for health and demographic variables.

NLC were highly associated (in unadjusted models) with multiple prevalent medical disorders, including depression, and cardiovascular, arthritis, and respiratory diseases. However, worse overall perceived health, more than any particular medical condition, was the strongest predictor of NLC of all health variables. Among specific medical conditions, and persisting after controlling for overall health, arthritis had a positive association with NLC. This association had been suggested by a previous small primary care study[5]. It is unclear whether it is the inflammatory state or damage to peripheral nerves that is responsible for this association with arthritis.

The association of cardiovascular symptoms and diseases with frequent NLC is complex. In unadjusted models, hypertension, heart failure and angina all were associated with more frequent NLC. Of note, in the stepwise regression, angina was a protective factor (those with angina were more likely to experience leg cramps in the logistic regression analyses but the direction was reversed in the stepwise regression). It is possible that those individuals with persistent angina use medications (e.g., nitrates) which are protective for NLC or avoid medications (e.g., long-acting beta agonists) which are thought to aggravate NLC[9].

The strongest association of a laboratory value to frequent NLC was with red blood cell count. In the stepwise regression for every increase of one million cells per microliter, the likelihood of moderate-severe NLC decreased by 57%. Interestingly, serum levels of iron and vitamins B12, B6, and E were not associated with NLC in adjusted models, each of which has been investigated as a treatment for frequent NLC[8].

An elevated HbA1c was also associated with frequent NLC, as were diabetes and fasting glucose (but not insulin), and the former variable was present after overall health was removed from the stepwise regression. Poorly controlled diabetes may be responsible for the association with NLC due to microvascular or neuropathic changes[14]. The absence of information on peripheral vascular disease and neuropathy in these NHANES cohorts makes interpretation of this association more difficult.

The most powerful single association with NLC is to self-reported "nocturnal leg jerks". The intent of this question in the NHANES surveys was to assess the prevalence of periodic limb movements of sleep (PLMS), a common sleep-related feature[15]. These movements involve rhythmic dorsiflexion of the ankle and at times, flexion at the knee, and last 0.5–10 seconds. They are more common with age and in those taking serotonergic reuptake inhibitors, and less common in African-Americans[16]. It is possible that such leg movements may provoke leg cramps in vulnerable individuals. Polysomnographic investigation into those with NLC may assist with this issue. Another possible explanation for this association is that NHANES respondents believed the question about "leg jerks" referred to NLC thus creating a spurious association between the two. To assess this association further, a Spearman correlation was computed. In this analysis, Rho = 0.35, suggesting that these constructs modestly overlap. For example, among those with moderate-severe leg jerks, 40% also experienced moderate-severe NLC as well, whereas 32% experienced no NLC at all.

Multiple sleep variables were associated with frequent NLC in partially controlled models. In the stepwise regression both difficulty falling asleep and non-restorative sleep were associated with frequent NLC, whereas difficulty maintaining sleep only emerged when leg jerks were removed from the model. This is surprising as NLC are usually associated with awakenings from sleep[17]. Although sleep medications were associated with frequent NLC, they were not a predictor in the stepwise regression suggesting that sleep disturbance, rather than sleep medications, is the relevant association. Similarly, the absence of indicators of sleep apnea, e.g., BMI, snoring, or snorting/gasping as predictors in the stepwise regression suggests that this sleep disorder is not an important independent contributor to NLC.

There are a number of limitations of this investigation. NLC were only assessed with one question, in a overall study not primarily addressing this symptom, limiting the reliability of the case ascertainment. Peripheral neuropathy and peripheral vascular disease were not assessed in these NHANES waves and thus the associations of these common neurological and vascular disorders with NLC could not be assessed. Activity level, another potential contributor to NLC, could also not be assessed due to inconsistent methods of evaluation in the two NHANES waves. Finally, restless legs syndrome (RLS) diagnosis was not assessed in either of these NHANES waves, and it is possible that misclassification of RLS as NLC may account for some of the medical and sleep comorbidities we observed with NLC, which are also present with RLS [18, 19].

The results of this first cross-sectional study based on a representative population demonstrate that NLC occur >15x/month in 6% of the population, more commonly with advancing age and in women, and are associated with poorer self-reported health, multiple sleep disturbance symptoms, abnormal laboratory tests, and multiple chronic medical conditions including depression. The extent to which such NLC are a marker or a cause of medical and psychiatric disability is unclear, and future research should address both their consequences for health as well as their underlying pathophysiology.

## Supporting information

S1 Table Associations with mild leg cramps in 2007–2008*. * using variables with p<0.05 in 2005–2006, ** ** adjusted for age, sex, education, BMI, employment, hypertension, diabetes, depression.
(DOCX)

S2 Table Associations with moderate-severe nocturnal leg cramps in 2007–2008, models adjusted for (1) age and sex and (2) age, sex, education, and body mass index*. * using variables with p<0.05 in 2005–2006.
(DOCX)

S3 Table Stepwise logistic regression results of associations between nocturnal leg cramps and all significant factors, excluding leg jerks and/or overall health (combined 2005–2008). (DOCX)
