# Supplementary material for: Nocturnal leg cramps: Prevalence and associations with demographics, sleep disturbance symptoms, medical conditions, and cardiometabolic risk factors
Source: PLoS One. 2017 Jun 6;12(6):e0178465. doi: 10.1371/journal.pone.0178465 (PMC5460850; doi:10.1371/journal.pone.0178465)
Supplement: S1 Table — * using variables with p<0.05 in 2005–2006, ** ** adjusted for age, sex, education, BMI, employment, hypertension, diabetes, depression. (DOCX) [file pone.0178465.s001.docx]

**S1 Table**. Associations with Mild Leg Cramps in 2007-2008*

|  |  | Unadjusted | | | Age and Sex | | | Age, Sex, Education, and Body Mass Index | | | Fully adjusted** | | | | | |
| --- | --- | --- | --- | --- | --- | --- | --- | --- | --- | --- | --- | --- | --- | --- | --- | --- |
| **Variable** |  | **OR** | **95% CI** | **P** | **OR** | **95% CI** | **P** | **OR** | **95% CI** | **P** | **OR** | **95% CI** | | | **P** | |
| **Demographics** | | | | | | | | | | | | | | | | |
| Age | Continuous | 1.02 | (1.01, 1.02) | <0.0001 |  | | |  | | | | | | | | |
| Age | ≥80 | 1.00 | Reference |  |  |  |  |  |  |  |  |  |  |  |  |  |
|  | 70-79 | 1.11 | (0.82, 1.49) | 0.500 |  |  |  |  |  |  |  |  |  |  |  |  |
|  | 60-69 | 1.16 | (0.86, 1.56) | 0.334 |  |  |  |  |  |  |  |  |  |  |  |  |
|  | 50-59 | 1.05 | (0.78, 1.41) | 0.748 |  |  |  |  |  |  |  |  |  |  |  |  |
|  | 40-49 | 0.70 | (0.52, 0.94) | 0.019 |  |  |  |  |  |  |  |  |  |  |  |  |
|  | 30-39 | 0.52 | (0.39, 0.70) | <0.0001 |  |  |  |  |  |  |  |  |  |  |  |  |
|  | 18-29 | 0.48 | (0.36, 0.65) | <0.0001 |  |  |  |  |  |  |  |  |  |  |  |  |
| Sex | Female | 1.14 | (0.98, 1.32) | 0.082 |  |  |  |  |  |  |  |  |  |  |  |  |
| Education | College Graduate | 1.00 | Reference |  |  |  |  |  |  |  |  |  |  |  |  |  |
|  | Some College | 1.49 | (1.20, 1.85) | 0.0003 | 1.50 | (1.20, 1.87) | 0.0003 |  |  |  |  |  |  |  |  |  |
|  | High School | 1.64 | (1.31, 2.05) | <0.0001 | 1.64 | (1.31, 2.06) | <0.0001 |  |  |  |  |  |  |  |  |  |
|  | Less Than High School | 1.34 | (1.07, 1.67) | 0.010 | 1.45 | (1.15, 1.81) | 0.0013 |  |  |  |  |  |  |  |  |  |
| Race/Ethnicity | Non-Hispanic White | 1.00 | Reference |  |  |  |  |  |  |  |  |  | | |  | |
|  | Black/African-American | 0.88 | (0.76, 1.04) | 0.131 | 1.04 | (0.88, 1.22) | 0.6739 | 0.97 | (0.82, 1.16) | 0.769 | 0.98 | (0.82, 1.19) | | | 0.8931 | |
|  | Hispanic/Latino | 0.90 | (0.76, 1.07) | 0.236 | 0.97 | (0.82, 1.16) | 0.7488 | 0.87 | (0.73, 1.05) | 0.16 | 0.86 | (0.71, 1.05) | | | 0.1308 | |
|  | Asian/Other | 0.96 | (0.68, 1.37) | 0.852 | 1.03 | (0.73, 1.46) | 0.867 | 1.15 | (0.80, 1.65) | 0.45 | 1.08 | (0.72, 1.64) | | | 0.6976 | |
| Marital Status | Married | 1.00 | Reference |  |  |  |  |  |  |  |  |  | | |  | |
|  | Divorced, Widowed, or Separated | 1.22 | (1.02, 1.46) | 0.033 | 1.05 | (0.86, 1.27) | 0.6458 | 1.02 | (0.83, 1.24) | 0.8749 | 0.99 | (0.81, 1.23) | | | 0.9498 | |
|  | Never Married | 0.64 | (0.51, 0.79) | <0.0001 | 0.86 | (0.69, 1.08) | 0.2062 | 0.86 | (0.68, 1.09) | 0.2135 | 0.88 | (0.68, 1.13) | | | 0.3185 | |
|  | Living With Partner | 0.66 | (0.48, 0.90) | 0.009 | 0.85 | (0.61, 1.17) | 0.3136 | 0.80 | (0.57, 1.12) | 0.2014 | 0.81 | (0.57, 1.16) | | | 0.2491 | |
| Employment | Unemployed | 1.35 | (1.17, 1.57) | <0.0001 | 1.05 | (0.89, 1.24) | 0.548 | 1.02 | (0.86, 1.22) | 0.778 | 0.97 | (0.81, 1.16) | | | 0.737 | |
| **Sleep** | | | | | | | | | | | | | | | | |
| Sleep Duration | Continuous | 0.91 | .869, 0.960) | 0.0004 | 0.90 | (0.86, 0.95) | 0.0001 | 0.93 | .883, 0.981) | 0.0071 | 0.94 | .893, 1.000) | | | 0.0491 | |
| Snoring | Never | 1.00 | Reference |  | 1.00 | Reference |  | 1.00 | Reference |  | 1.00 | Reference | | |  | |
|  | Rarely (1/week) | 1.29 | (1.02, 1.64) | 0.032 | 1.26 | (0.99, 1.60) | 0.0584 | 1.23 | (0.96, 1.57) | 0.0989 | 1.23 | (0.95, 1.60) | | | 0.1106 | |
|  | Occasionally (3-4/week) | 1.61 | (1.29, 2.02) | <0.0001 | 1.51 | (1.20, 1.89) | 0.0004 | 1.43 | (1.13, 1.80) | 0.003 | 1.51 | (1.18, 1.93) | | | 0.0012 | |
|  | Frequently (≥5/week) | 1.64 | (1.34, 2.00) | <0.0001 | 1.54 | (1.26, 1.89) | <0.0001 | 1.30 | (1.05, 1.62) | 0.018 | 1.33 | (1.06, 1.69) | | | 0.0156 | |
| Snorting/Gasping | Never | 1.00 | Reference |  | 1.00 | Reference |  | 1.00 | Reference |  | 1.00 | Reference | | |  | |
|  | Rarely (1/week) | 1.76 | (1.36, 2.29) | <0.0001 | 1.74 | (1.34, 2.27) | <0.0001 | 1.65 | (1.26, 2.17) | 0.0003 | 1.49 | (1.12, 1.97) | | | 0.0058 | |
|  | Occasionally (3-4/week) | 1.54 | (1.14, 2.08) | 0.004 | 1.49 | (1.10, 2.01) | 0.0092 | 1.44 | (1.06, 1.96) | 0.0212 | 1.30 | (0.94, 1.81) | | | 0.1177 | |
|  | Frequently (≥5/week) | 1.23 | (0.88, 1.72) | 0.225 | 1.16 | (0.83, 1.62) | 0.3808 | 0.96 | (0.68, 1.36) | 0.8356 | 0.86 | (0.59, 1.23) | | | 0.403 | |
| Difficulty Falling Asleep | None | 1.00 | Reference |  | 1.00 | Reference |  | 1.00 | Reference |  | 1.00 | Reference | | |  | |
|  | Mild (<15/month) | 1.88 | (1.60, 2.23) | <0.0001 | 1.89 | (1.60, 2.23) | <0.0001 | 1.98 | (1.66, 2.36) | <0.0001 | 1.96 | (1.63, 2.35) | | | <0.0001 | |
|  | Moderate-Severe | 2.36 | (1.91, 2.91) | <0.0001 | 2.41 | (1.94, 2.98) | <0.0001 | 2.38 | (1.91, 2.97) | <0.0001 | 2.22 | (1.75, 2.83) | | | <0.0001 | |
| Difficulty Maintaining Sleep | None | 1.00 | Reference |  | 1.00 | Reference |  | 1.00 | Reference |  | 1.00 | Reference | | |  | |
|  | Mild (<15/month) | 1.96 | (1.65, 2.33) | <0.0001 | 1.91 | (1.61, 2.27) | <0.0001 | 1.96 | (1.64, 2.35) | <0.0001 | 1.94 | (1.61, 2.35) | | | <0.0001 | |
|  | Moderate-Severe | 2.22 | (1.80, 2.72) | <0.0001 | 2.13 | (1.72, 2.62) | <0.0001 | 2.08 | (1.68, 2.58) | <0.0001 | 1.96 | (1.55, 2.48) | | | <0.0001 | |
| Non-Restorative Sleep | None | 1.00 | Reference |  | 1.00 | Reference |  | 1.00 | Reference |  | 1.00 | Reference | | |  | |
|  | Mild (<15/month) | 1.55 | (1.30, 1.84) | <0.0001 | 1.72 | (1.44, 2.06) | <0.0001 | 1.71 | (1.42, 2.05) | <0.0001 | 1.62 | (1.34, 1.98) | | | <0.0001 | |
|  | Moderate-Severe | 2.09 | (1.71, 2.54) | <0.0001 | 2.49 | (2.03, 3.04) | <0.0001 | 2.43 | (1.97, 2.99) | <0.0001 | 2.08 | (1.65, 2.63) | | | <0.0001 | |
| Daytime Sleepiness | None | 1.00 | Reference |  | 1.00 | Reference |  | 1.00 | Reference |  | 1.00 | Reference | | |  | |
|  | Mild (<15/month) | 1.71 | (1.45, 2.02) | <0.0001 | 1.86 | (1.57, 2.20) | <0.0001 | 1.84 | (1.54, 2.19) | <0.0001 | 1.76 | (1.46, 2.11) | | | <0.0001 | |
|  | Moderate-Severe | 1.94 | (1.57, 2.41) | <0.0001 | 2.21 | (1.78, 2.74) | <0.0001 | 2.08 | (1.67, 2.61) | <0.0001 | 1.78 | (1.39, 2.28) | | | <0.0001 | |
| Use of Sleep Medication | None | 1.00 | Reference |  | 1.00 | Reference |  | 1.00 | Reference |  | 1.00 | Reference | | |  | |
|  | Mild (<15/month) | 1.70 | (1.34, 2.15) | <0.0001 | 1.64 | (1.28, 2.08) | 0.0001 | 1.65 | (1.29, 2.11) | 0.0001 | 1.60 | (1.24, 2.08) | | | 0.0004 | |
|  | Moderate-Severe | 1.60 | (1.23, 2.07) | 0.0004 | 1.40 | (1.07, 1.82) | 0.0137 | 1.34 | (1.02, 1.77) | 0.0357 | 1.26 | (0.93, 1.71) | | | 0.135 | |
| Leg Jerks | None | 1.00 | Reference |  | 1.00 | Reference |  | 1.00 | Reference |  | 1.00 | Reference | | |  | |
|  | Mild (<15/month) | 4.36 | (3.61, 5.27) | <0.0001 | 4.67 | (3.84, 5.67) | <0.0001 | 4.67 | (3.81, 5.71) | <0.0001 | 4.54 | (3.67, 5.62) | | | <0.0001 | |
|  | Moderate-Severe | 2.72 | (1.96, 3.77) | <0.0001 | 2.75 | (1.97, 3.83) | <0.0001 | 2.77 | (1.97, 3.88) | <0.0001 | 2.62 | (1.85, 3.71) | | | <0.0001 | |
| **Medical History** | | | | | | | | | | | | | | | | |
| Smoking | Current Smoker | 1.08 | (0.91, 1.28) | 0.399 | 1.27 | (1.07, 1.53) | 0.008 | 1.15 | (0.95, 1.41) | 0.145 | 1.15 | (0.95, 1.41) | | | 0.145 | |
| Overall Health | Excellent | 1.00 | Reference |  | 1.00 | Reference |  | 1.00 | Reference |  | 1.00 | Reference | | |  | |
|  | Very Good | 1.28 | (0.99, 1.66) | 0.060 | 1.22 | (0.94, 1.58) | 0.1381 | 1.22 | (0.93, 1.59) | 0.1512 | 1.24 | (0.93, 1.64) | | | 0.1394 | |
|  | Good | 1.80 | (1.41, 2.31) | <0.0001 | 1.67 | (1.30, 2.14) | 0.0001 | 1.61 | (1.25, 2.09) | 0.0003 | 1.59 | (1.21, 2.09) | | | 0.0009 | |
|  | Fair | 2.75 | (2.10, 3.60) | <0.0001 | 2.45 | (1.87, 3.22) | <0.0001 | 2.38 | (1.79, 3.17) | <0.0001 | 2.33 | (1.71, 3.18) | | | <0.0001 | |
|  | Poor | 4.40 | (3.03, 6.40) | <0.0001 | 3.59 | (2.45, 5.28) | <0.0001 | 3.74 | (2.51, 5.58) | <0.0001 | 3.43 | (2.18, 5.37) | | | <0.0001 | |
| Hypertension |  | 1.57 | (1.35, 1.83) | <0.0001 | 1.22 | (1.03, 1.45) | 0.0245 | 1.07 | (0.89, 1.29) | 0.4597 |  | | | | | |
| Diabetes |  | 1.86 | (1.50, 2.31) | <0.0001 | 1.49 | (1.19, 1.87) | 0.0006 | 1.28 | (1.01, 1.63) | 0.043 |  |  |  |  |  |  |
| Heart Failure |  | 1.69 | (1.16, 2.48) | 0.007 | 1.17 | (0.79, 1.75) | 0.4333 | 1.12 | (0.74, 1.67) | 0.5978 | 0.98 | .632, 1.53) | | | 0.9436 | |
| Angina |  | 1.30 | (0.84, 2.00) | 0.235 | 0.90 | (0.59, 1.41) | 0.6698 | 0.84 | (0.54, 1.33) | 0.4679 | 0.78 | (0.48, 1.28) | | | 0.3343 | |
| Heart Attack |  | 1.55 | (1.11, 2.17) | 0.009 | 1.09 | (0.78, 1.54) | 0.6083 | 1.07 | (0.76, 1.53) | 0.6861 | 1.09 | (0.75, 1.58) | | | 0.6475 | |
| Arthritis |  | 2.29 | (1.94, 2.69) | <0.0001 | 1.86 | (1.56, 2.23) | <0.0001 | 1.71 | (1.42, 2.06) | <0.0001 | 1.65 | (1.36, 2.02) | | | <0.0001 | |
| Respiratory Disease |  | 2.20 | (1.69, 2.86) | <0.0001 | 1.88 | (1.44, 2.45) | <0.0001 | 1.76 | (1.35, 2.31) | <0.0001 | 1.59 | (1.20, 2.11) | | | 0.001 | |
| Thyroid Disease |  | 1.74 | (1.37, 2.21) | <0.0001 | 1.42 | (1.10, 1.83) | 0.0067 | 1.47 | (1.13, 1.91) | 0.0041 | 1.43 | (1.09, 1.87) | | | 0.0105 | |
| Asthma |  | 1.36 | (1.04, 1.77) | 0.025 | 1.35 | (1.03, 1.76) | 0.0269 | 1.19 | (0.91, 1.57) | 0.205 | 1.10 | (0.83, 1.47) | | | 0.5098 | |
| Depression | PHQ Score | 1.06 | (1.04, 1.08) | <0.0001 | 1.06 | (1.04, 1.08) | <0.0001 | 1.06 | (1.04, 1.08) | <0.0001 |  | | | | | |
|  | PHQ Score - Sleep | 1.06 | (1.04, 1.09) | <0.0001 | 1.07 | (1.05, 1.09) | <0.0001 | 1.06 | (1.04, 1.08) | <0.0001 |  |  |  |  |  |  |
| Diuretics |  | 1.74 | (1.42, 2.14) | <0.0001 | 1.27 | (1.02, 1.58) | 0.034 | 1.11 | (0.88, 1.40) | 0.369 | 1.15 | | (0.87, 1.44) | 0.399 | |  |
| Long-acting beta agonists |  | 3.89 | (1.12, 13.53) | 0.032 | 3.20 | (0.90, 11.32) | 0.072 | 2.45 | (0.70, 8.59) | 0.162 | 2.19 | | (0.64, 7.52) | 0.210 | |  |
| **Objective Health**  **Variables** | | | | | | | | | | | | | | | | |
| Body Mass Index |  | 1.03 | (1.02, 1.04) | <0.0001 | 1.03 | (1.01, 1.04) | <0.0001 |  | | |  | | | | | |
| Calcium | Log | 0.54 | (0.08, 3.88) | 0.548 | 0.77 | .109, 5.515) | 0.8001 | 0.83 | (0.11, 6.22) | 0.8607 | 0.68 | (0.08, 5.66) | | | 0.7276 | |
| Folate | Log | 0.90 | (0.80, 1.01) | 0.076 | 0.95 | (0.84, 1.07) | 0.381 | 1.01 | (0.89, 1.14) | 0.923 | 1.02 | (0.89, 1.17) | | | 0.764 | |
| C-Reactive Protein | Log | 1.19 | (1.12, 1.26) | <0.0001 | 1.15 | (1.08, 1.23) | <0.0001 | 1.10 | (1.02, 1.18) | 0.0098 | 1.12 | (1.04, 1.20) | | | 0.0028 | |
| HbA1c% |  | 1.28 | (1.19, 1.39) | <0.0001 | 1.16 | (1.07, 1.26) | 0.0002 | 1.10 | (1.01, 1.20) | 0.0211 | 1.08 | (0.98, 1.19) | | | 0.1034 | |
| Glucose | Log | 2.29 | (1.43, 3.66) | 0.0006 | 1.71 | (1.04, 2.79) | 0.0328 | 1.40 | (0.83, 2.36) | 0.2071 | 1.13 | (0.62, 2.04) | | | 0.6967 | |
| Cadmium | Log | 1.17 | (1.02, 1.33) | 0.021 | 1.08 | (0.94, 1.24) | 0.301 | 1.08 | (0.94, 1.24) | 0.302 | 1.05 | (0.90, 1.22) | | | 0.521 | |
| White Blood Cell Count |  | 1.06 | (1.02, 1.09) | 0.0016 | 1.07 | (1.03, 1.11) | 0.0003 | 1.04 | (1.00, 1.08) | 0.0433 | 1.02 | (0.99, 1.06) | | | 0.197 | |
| Red Blood Cell Count |  | 0.78 | (0.67, 0.92) | 0.003 | 0.91 | .760, 1.110) | 0.3773 | 0.85 | .705, 1.039) | 0.1154 | 0.87 | (0.71, 1.07) | | | 0.191 | |

*** using variables with p<0.05 in 2005-2006**

**** ** adjusted for age, sex, education, BMI, employment, hypertension, diabetes, depression**
