# Supplementary material for: Nocturnal leg cramps: Prevalence and associations with demographics, sleep disturbance symptoms, medical conditions, and cardiometabolic risk factors
Source: PLoS One. 2017 Jun 6;12(6):e0178465. doi: 10.1371/journal.pone.0178465 (PMC5460850; doi:10.1371/journal.pone.0178465)
Supplement: S2 Table — * using variables with p<0.05 in 2005–2006. (DOCX) [file pone.0178465.s002.docx]

**S2 Table**. Associations with Moderate-Severe Nocturnal Leg Cramps in 2007-2008, Models Adjusted for (1) Age and Sex and (2) Age, Sex, Education, and Body Mass Index*

|  |  | Age and Sex | | | Age, Sex, Education, and BMI | | |
| --- | --- | --- | --- | --- | --- | --- | --- |
| **Variable** |  | **OR** | **95% CI** | **P** | **OR** | **95% CI** | **P** |
| **Demographics** | | | | | | | |
| Education | College Graduate | 1.00 | Reference |  |  | | |
|  | Some College | 4.88 | (3.14, 7.59) | <0.0001 |  |  |  |
|  | High School | 2.92 | (1.81, 4.73) | <0.0001 |  |  |  |
|  | Less Than High School | 2.49 | (1.53, 4.03) | 0.0002 |  |  |  |
| Race/Ethnicity | Non-Hispanic White | 1.00 | Reference |  |  |  |  |
|  | Black/African-American | 1.24 | (0.94, 1.64) | 0.1205 | 0.824 | (0.61, 1.12) | 0.2106 |
|  | Hispanic/Latino | 0.87 | (0.63, 1.20) | 0.3850 | 0.645 | (0.46, 0.92) | 0.0132 |
|  | Asian/Other | 1.00 | (0.57, 1.75) | 0.9988 | 1.174 | (0.66, 2.08) | 0.5838 |
| Marital Status | Married | 1.00 | Reference |  |  |  |  |
|  | Divorced, Widowed, or Separated | 1.51 | (1.12, 2.03) | 0.0071 | 1.37 | (1.01, 1.85) | 0.0458 |
|  | Never Married | 0.94 | (0.60, 1.46) | 0.7842 | 0.86 | (0.54, 1.38) | 0.5403 |
|  | Living With Partner | 1.95 | (1.17, 3.24) | 0.0108 | 1.63 | (0.97, 2.72) | 0.0632 |
| Employment | Unemployed | 1.96 | (1.46, 2.63) | <0.0001 | 1.63 | (1.20, 2.21) | 0.0016 |
| **Sleep** | | | | | | | |
| Sleep Duration | Continuous | 0.695 | (0.63, 0.77) | <0.0001 | 0.730 | (0.66, 0.80) | <0.0001 |
| Snoring | Never | 1.00 | Reference |  | 1.00 | Reference |  |
|  | Rarely (1/week) | 1.02 | (0.64, 1.63) | 0.9289 | 1.02 | (0.63, 1.66) | 0.9207 |
|  | Occasionally (3-4/week) | 1.45 | (0.97, 2.17) | 0.0691 | 1.4 | (0.93, 2.12) | 0.1095 |
|  | Frequently (≥5/week) | 2.29 | (1.61, 3.26) | <0.0001 | 1.86 | (1.29, 2.69) | 0.001 |
| Snorting/Gasping | Never | 1.00 | Reference |  | 1.00 | Reference |  |
|  | Rarely (1/week) | 2 | (1.25, 3.19) | 0.0036 | 1.98 | (1.23, 3.18) | 0.0051 |
|  | Occasionally (3-4/week) | 2.7 | (1.76, 4.13) | <0.0001 | 2.51 | (1.61, 3.91) | 0.0001 |
|  | Frequently (≥5/week) | 3.67 | (2.32, 5.82) | <0.0001 | 3.07 | (1.90, 4.97) | <0.0001 |
| Difficulty Falling Asleep | None | 1.00 | Reference |  | 1.00 | Reference |  |
|  | Mild (<15/month) | 1.82 | (1.28, 2.59) | 0.0009 | 2.13 | (1.47, 3.07) | 0.0001 |
|  | Moderate-Severe | 5.67 | (3.96, 8.12) | <0.0001 | 5.71 | (3.93, 8.29) | <0.0001 |
| Difficulty Maintaining Sleep | None | 1.00 | Reference |  | 1.00 | Reference |  |
|  | Mild (<15/month) | 1.82 | (1.27, 2.61) | 0.0012 | 2.17 | (1.49, 3.15) | <0.0001 |
|  | Moderate-Severe | 6.18 | (4.31, 8.86) | <0.0001 | 6.68 | (4.59, 9.73) | <0.0001 |
| Non-Restorative Sleep | None | 1.00 | Reference |  | 1.00 | Reference |  |
|  | Mild (<15/month) | 2 | (1.35, 2.96) | 0.0006 | 2.32 | (1.54, 3.48) | 0.0001 |
|  | Moderate-Severe | 7.95 | 5.46, 11.58) | <0.0001 | 8.86 | (6.02, 13.06) | <0.0001 |
| Daytime Sleepiness | None | 1.00 | Reference |  | 1.00 | Reference |  |
|  | Mild (<15/month) | 2.83 | (1.99, 4.03) | <0.0001 | 3.18 | (2.23, 4.55) | <0.0001 |
|  | Moderate-Severe | 10 | 6.97, 14.40) | <0.0001 | 10.04 | (6.96, 14.48) | <0.0001 |
| Use of Sleep Medication | None | 1.00 | Reference |  | 1.00 | Reference |  |
|  | Mild (<15/month) | 1.74 | (1.19, 2.54) | 0.0044 | 1.88 | (1.26, 2.80) | 0.0019 |
|  | Moderate-Severe | 4.1 | (2.93, 5.72) | <0.0001 | 4.26 | (3.00, 6.05) | <0.0001 |
| Leg Jerks | None | 1.00 | Reference |  | 1.00 | Reference |  |
|  | Mild (<15/month) | 3.31 | (2.27, 4.83) | <0.0001 | 3.28 | (2.23, 4.83) | <0.0001 |
|  | Moderate-Severe | 20.7 | 4.58, 29.64) | <0.0001 | 20.04 | (3.77, 29.15) | <0.0001 |
| **Medical**  **History** | | | | | | | |
| Smoking | Current Smoker | 2.42 | (1.80, 3.26) | <0.0001 | 1.67 | (1.18, 2.37) | 0.004 |
| Overall Health | Excellent | 1.00 | Reference |  | 1.00 | Reference |  |
|  | Very Good | 0.95 | (0.50, 1.81) | 0.8758 | 0.92 | (0.47, 1.78) | 0.8051 |
|  | Good | 1.96 | (1.10, 3.50) | 0.0225 | 1.71 | (0.93, 3.14) | 0.0829 |
|  | Fair | 7.73 | 4.35, 13.72) | <0.0001 | 6.3 | (3.39, 11.70) | <0.0001 |
|  | Poor | 13.1 | 6.89, 25.17) | <0.0001 | 10.2 | (5.00, 20.91) | <0.0001 |
| Hypertension | Yes | 2.29 | (1.72, 3.05) | <0.0001 | 2.05 | (1.53, 2.76) | <0.0001 |
| Diabetes | Yes | 2.43 | (1.74, 3.39) | <0.0001 | 1.86 | (1.30, 2.66) | 0.0008 |
| Heart Failure | Yes | 2.68 | (1.62, 4.44) | 0.0001 | 2.27 | (1.32, 3.89) | 0.0029 |
| Angina | Yes | 2.08 | (0.20, 3.66) | 0.0093 | 1.89 | (1.06, 3.38) | 0.03 |
| Heart Attack | Yes | 3.06 | (1.96, 4.75) | <0.0001 | 2.64 | (1.68, 4.16) | <0.0001 |
| Arthritis | Yes | 5.07 | (3.71, 6.92) | <0.0001 | 4.59 | (3.32, 6.33) | <0.0001 |
| Respiratory Disease | Yes | 4.09 | (2.91, 5.76) | <0.0001 | 3.44 | (2.43, 4.86) | <0.0001 |
| Thyroid Disease | Yes | 1.4 | (0.94, 2.08) | 0.0947 | 1.62 | (1.08, 2.44) | 0.021 |
| Asthma | Yes | 3.53 | (2.50, 5.00) | <0.0001 | 3.14 | (2.21, 4.46) | <0.0001 |
| Depression | PHQ Score | 1.2 | (1.17, 1.23) | <0.0001 | 1.18 | (1.15, 1.21) | <0.0001 |
|  | PHQ Score - Sleep | 1.22 | (1.18, 1.26) | <0.0001 | 1.2 | (1.16, 1.23) | <0.0001 |
| Diuretics | Yes | 1.67 | (1.15, 2.41) | 0.007 | 1.41 | (0.95, 2.08) | 0.086 |
| **Objective**  **Health**  **Variables** | | | | | | | |
| Body Mass Index |  | 1.04 | (1.02, 1.06) | <0.0001 |  | | |
| Calcium | Log | 0.57 | (0.03, 11.50) | 0.7153 | 1.31 | (0.06, 28.67) | 0.8625 |
| Folate | Log | 0.73 | (0.58, 0.91) | 0.005 | 0.84 | (0.67, 1.06) | 0.145 |
| C-Reactive Protein | Log | 1.27 | (1.14, 1.41) | <0.0001 | 1.16 | (1.02, 1.31) | 0.0214 |
| HbA1c% |  | 1.28 | (1.16, 1.42) | <0.0001 | 1.16 | (1.04, 1.30) | 0.0086 |
| Glucose | Log | 2.87 | (1.57, 5.22) | 0.0006 | 2.26 | (1.19, 4.30) | 0.0128 |
| Cadmium | Log | 1.26 | (0.99, 1.60) | 0.059 | 1.18 | (0.93, 1.50) | 0.166 |
| White Blood Cell Count |  | 1.11 | (1.05, 1.17) | 0.0002 | 1.07 | (1.02, 1.12) | 0.01 |
| Red Blood Cell Count |  | 0.84 | (0.63, 1.13) | 0.2606 | 0.73 | (0.55, 0.99) | 0.0391 |

*** using variables with p<0.05 in 2005-2006**
