# Supplementary material for: Nocturnal leg cramps: Prevalence and associations with demographics, sleep disturbance symptoms, medical conditions, and cardiometabolic risk factors
Source: PLoS One. 2017 Jun 6;12(6):e0178465. doi: 10.1371/journal.pone.0178465 (PMC5460850; doi:10.1371/journal.pone.0178465)
Supplement: S3 Table — (DOCX) [file pone.0178465.s003.docx]

**S3 Table**. Stepwise Logistic Regression Results of Associations between Nocturnal Leg Cramps and All Significant Factors, Excluding Leg Jerks and/or Overall Health (Combined 2005-2008)

|  | | **Model Change** | **Mild  Nocturnal Leg Cramps** | | | | **Moderate-Severe  Nocturnal Leg Cramps** | | |
| --- | --- | --- | --- | --- | --- | --- | --- | --- | --- |
| **Variable** | | **P** | **OR** | **95% CI** | **p** | **OR** | | **95% CI** | **p** |
| **Excluding Leg Jerks** | | | | | | | | | |
| Overall Health | Very Good | <0.0001 | 0.758 | (0.559, 1.027) | 0.073 | 0.621 | | (0.272, 1.415) | 0.257 |
|  | Good |  | 0.788 | (0.618, 1.006) | 0.056 | 0.841 | | (0.488, 1.45) | 0.533 |
|  | Fair |  | 0.953 | (0.765, 1.188) | 0.67 | 0.614 | | (0.383, 0.984) | 0.043 |
|  | Poor |  | 2.062 | (1.533, 2.773) | <0.0001 | 3.849 | | (2.308, 6.421) | <0.0001 |
| Arthritis | Yes | <0.0001 | 1.802 | (1.294, 2.51) | <0.0001 | 3.807 | | (2.127, 6.814) | <0.0001 |
| Difficulty Falling Asleep | Mild | <0.0001 | 1.553 | (1.117, 2.161) | 0.009 | 3.158 | | (1.695, 5.886) | <0.0001 |
|  | Moderate-Severe |  | 2.283 | (1.453, 3.587) | <0.0001 | 3.196 | | (1.508, 6.772) | 0.002 |
| Non-Restorative Sleep | Mild | 0.0010 | 1.264 | (0.896, 1.783) | 0.181 | 2.321 | | (1.219, 4.418) | 0.01 |
|  | Moderate-Severe |  | 2.081 | (1.386, 3.124) | <0.0001 | 4.347 | | (2.141, 8.824) | <0.0001 |
| Age | Years | 0.0001 | 1.018 | (1.008, 1.028) | <0.0001 | 1.021 | | (1.001, 1.042) | 0.039 |
| Difficulty Maintaining Sleep | Mild | 0.0120 | 1.454 | (1.026, 2.06) | 0.035 | 1.258 | | (0.697, 2.273) | 0.446 |
|  | Moderate-Severe |  | 0.897 | (0.566, 1.421) | 0.643 | 1.975 | | (0.998, 3.907) | 0.051 |
| Education | Some College | 0.0126 | 1.694 | (1.097, 2.614) | 0.017 | 2.806 | | (1.257, 6.266) | 0.012 |
|  | High School |  | 1.72 | (1.155, 2.562) | 0.008 | 1.453 | | (0.618, 3.412) | 0.391 |
|  | Less than High School |  | 1.522 | (1.028, 2.255) | 0.036 | 1.357 | | (0.554, 3.326) | 0.504 |
| Red Blood Cell Count |  | 0.0092 | 1.146 | (0.851, 1.544) | 0.369 | 0.487 | | (0.272, 0.872) | 0.015 |
| Asthma | Yes | 0.0462 | 0.841 | (0.513, 1.381) | 0.495 | 2.053 | | (1.001, 4.21) | 0.05 |
| Angina | Yes | 0.0419 | 0.393 | (0.156, 0.99) | 0.048 | 0.324 | | (0.115, 0.913) | 0.033 |
| HBA1c% |  | 0.0440 | 1.169 | (1.012, 1.35) | 0.034 | 1.227 | | (1.003, 1.501) | 0.047 |
| **Excluding Leg Jerks and Overall Health** | | | | | | | | | |
| Arthritis | Yes | <0.0001 | 1.806 | (1.303, 2.503) | <0.0001 | 3.586 | | (2.024, 6.353) | <0.0001 |
| Difficulty Falling Asleep | Mild | <0.0001 | 1.538 | (1.112, 2.127) | 0.009 | 2.81 | | (1.534, 5.148) | 0.001 |
|  | Moderate-Severe |  | 2.303 | (1.457, 3.64) | <0.0001 | 2.785 | | (1.289, 6.015) | 0.009 |
| HBA1c% |  | <0.0001 | 1.216 | (1.063, 1.391) | 0.004 | 1.292 | | (1.071, 1.559) | 0.008 |
| Non-Restorative Sleep | Mild | <0.0001 | 1.22 | (0.868, 1.714) | 0.252 | 2.15 | | (1.133, 4.08) | 0.019 |
|  | Moderate-Severe |  | 1.967 | (1.307, 2.96) | 0.001 | 3.586 | | (1.763, 7.295) | <0.0001 |
| Education | Some College | <0.0001 | 2.057 | (1.359, 3.113) | 0.001 | 3.892 | | (1.843, 8.222) | <0.0001 |
|  | High School |  | 1.842 | (1.243, 2.729) | 0.002 | 1.547 | | (0.689, 3.476) | 0.29 |
|  | Less than High School |  | 1.544 | (1.046, 2.28) | 0.029 | 1.377 | | (0.584, 3.246) | 0.464 |
| Age | Years | 0.0004 | 1.018 | (1.009, 1.028) | <0.0001 | 1.023 | | (1.004, 1.043) | 0.02 |
| Depression | Score | 0.0013 | 1.054 | (1.005, 1.105) | 0.029 | 1.11 | | (1.038, 1.188) | 0.002 |
| Difficulty Maintaining Sleep | Mild | 0.0129 | 1.412 | (1, 1.994) | 0.05 | 1.213 | | (0.682, 2.157) | 0.512 |
|  | Moderate-Severe |  | 0.899 | (0.566, 1.428) | 0.652 | 1.981 | | (1.005, 3.903) | 0.048 |
| Red Blood Cell Count |  | 0.0171 | 1.167 | (0.874, 1.558) | 0.295 | 0.538 | | (0.302, 0.958) | 0.035 |
| Asthma | Yes | 0.0266 | 0.92 | (0.562, 1.505) | 0.739 | 2.282 | | (1.139, 4.571) | 0.02 |
